# Vector-borne transmission of *Trypanosoma cruzi* among captive Neotropical primates in a Brazilian zoo

**DOI:** 10.1186/s13071-016-1334-7

**Published:** 2016-01-26

**Authors:** Thaís Tâmara Castro Minuzzi-Souza, Nadjar Nitz, Monique Britto Knox, Filipe Reis, Luciana Hagström, César A. Cuba Cuba, Mariana Machado Hecht, Rodrigo Gurgel-Gonçalves

**Affiliations:** Laboratório de Parasitologia Médica e Biologia de Vetores, Área de Patologia, Faculdade de Medicina, Universidade de Brasília, Campus Universitário Darcy Ribeiro, Asa Norte, Brasília, 70910-900 Distrito Federal Brazil; Laboratório Interdisciplinar de Biociências, Faculdade de Medicina, Universidade de Brasília, Brasília, Federal District Brazil; Diretoria de Vigilância Ambiental do Distrito Federal, Secretaria de Saúde, Brasília, Federal District Brazil; Fundação Jardim Zoológico de Brasília, Brasília, Federal District Brazil

**Keywords:** *Trypanosoma cruzi*, Neotropical primates, *Panstrongylus megistus*, Brasília, Zoo

## Abstract

**Background:**

Neotropical primates are important sylvatic hosts of *Trypanosoma cruzi*, the etiological agent of Chagas disease. Infection is often subclinical, but severe disease has been described in both free-ranging and captive primates. *Panstrongylus megistus*, a major *T. cruzi* vector, was found infesting a small-primate unit at Brasília zoo (ZooB), Brazil. ZooB lies close to a gallery-forest patch where *T. cruzi* circulates naturally. Here, we combine parasitological and molecular methods to investigate a focus of *T. cruzi* infection involving triatomine bugs and Neotropical primates at a zoo located in the Brazilian Savannah.

**Methods:**

We assessed *T. cruzi* infection in vectors using optical microscopy (*n* = 34) and nested PCR (*n* = 50). We used quantitative PCR (qPCR) to examine blood samples from 26 primates and necropsy samples from two primates that died during the study. We determined parasite lineages in five vectors and two primates by comparing glucose-6-phosphate isomerase (G6pi) gene sequences.

**Results:**

*Trypanosoma cruzi* was found in 44 vectors and 17 primates (six genera and eight species); one *Mico chrysoleucus* and one *Saguinus niger* had high parasitaemias. *Trypanosoma cruzi* DNA was detected in three primates born to qPCR-negative mothers at ZooB and in the two dead specimens. One *Callithrix geoffroyi* became qPCR-positive over a two-year follow-up. All G6pi sequences matched *T. cruzi* lineage TcI.

**Conclusions:**

Our findings strongly suggest vector-borne *T. cruzi* transmission within a small-primate unit at ZooB – with vectors, and perhaps also parasites, presumably coming from nearby gallery forest. Periodic checks for vectors and parasites would help eliminate *T. cruzi* transmission foci in captive-animal facilities. This should be of special importance for captive-breeding programs involving endangered mammals, and would reduce the risk of accidental *T. cruzi* transmission to keepers and veterinarians.

**Electronic supplementary material:**

The online version of this article (doi:10.1186/s13071-016-1334-7) contains supplementary material, which is available to authorized users.

## Background

About one third of all protozoan parasite species detected in non-human primates can also infect humans; *Trypanosoma cruzi*, the etiological agent of Chagas disease, is among the most epidemiologically relevant ones [1–3], [see also http://www.mammalparasites.org]. Chagas disease is endemic throughout Latin America, where about six million people are infected with *T. cruzi* [4–6]. *Trypanosoma cruzi*, a parasite of mammals, is transmitted primarily through the faeces of blood-sucking triatomine bugs; less often, infection can be acquired congenitally, through blood transfusion or organ or bone marrow transplantation, by consuming contaminated food or beverages, or accidentally in the laboratory [4, 5]. Seven highly diverse *T. cruzi* lineages circulate among mammals (at least eight orders and over 50 genera) and triatomines (over 140 species) in all continental American countries except Canada [3, 7, 8].

Carlos Chagas was the first to describe experimental (*Callithrix* spp.) and natural (*Saimiri sciureus*) *T. cruzi* infections in primates [9, 10]. Since then, the infection has been recorded in free-ranging individuals of 12 genera and over 30 species in all four Neotropical primate families – tamarins, marmosets, pygmy marmosets, squirrel monkeys and capuchins (Cebidae); titis, sakis and uakaris (Pitheciidae); night monkeys (Aotidae); and spider and howler monkeys (Atelidae) [3], [see also www.mammalparasites.org]. Lion tamarins (*Leontopithecus* spp.) can sustain long-lasting infections, often with high parasitaemias but with a relatively mild clinical picture, in the Brazilian Atlantic forest [3, 11–14].

*Trypanosoma cruzi* infections have also been reported in captive Neotropical primates. In the USA, *T. cruzi* was found in *Saimiri boliviensis* imported from Latin America [15]. *Trypanosoma cruzi* was detected in captive, wild-born *Callithrix penicillata*, *Cebuella pygmaea*, *Saguinus imperator*, and *S. fuscicollis* kept at the Brazilian National Primate Centre in Pará state [16]. Anti-*T. cruzi* antibodies were detected in 40 out of 198 captive primates (*Cacajao*, *Callicebus*, *Callithrix*, *Cebus*, *Chiropotes*, *Leontopithecus*, and *Saguinus*) from the Primatology Centre of Rio de Janeiro, Brazil, where transmission mediated by *Panstrongylus megistus* was suspected [17]. Captive Old World primates, including lemurs, macaques, baboons, and chimpanzees, can also become naturally infected with *T. cruzi* and may develop severe Chagas disease [18–22].

Infection of captive primates with *T. cruzi* is relevant in several respects. First, *T. cruzi* can kill valuable specimens including those belonging to endangered species; second, infection of laboratory primates can distort the results of animal-based research aimed at other ends; third, infected individuals in translocation-reintroduction programs can contribute to the spread of the parasite among free-ranging populations; finally, and importantly, infection can result in accidental transmission of the parasite to primate keepers, handlers, or veterinarians. Here, we combine parasitological and molecular methods to investigate a focus of *T. cruzi* infection involving triatomine bugs and Neotropical primates at a zoo located in the Brazilian Cerrado, where *T. cruzi* circulates extensively among wildlife and native vectors.

## Methods

### Ethics statement

This study was approved by the institutional review board of the Institute of Biological Sciences, University of Brasília, Brazil (CEUA-UnB No. 155506/2013).

### Study site

Brasília, Brazil’s capital city, lies within the Cerrado eco-region, a mosaic of savannahs, dry forests/shrubs, and gallery forests originally covering most of central Brazil. Enzootic *T. cruzi* cycles are common in the Cerrado [3]. Brasília zoo (ZooB; 15°51’00”S, 47°56’20”W) spans ~140 hectares; to the south and south-west, it is adjacent to a protected, and hence relatively well-preserved, gallery-forest patch (~490 hectares) where *T. cruzi* infection has been recorded in *Didelphis albiventris* [23]. The small-primate unit at ZooB has four lodgings, each with a wire-mesh–fenced outdoor area and a masonry room with a wooden-box shelter. In 2012, ZooB keepers detected a triatomine bug colony in the small-primate unit.

### *Trypanosoma cruzi* in triatomine bugs

Triatomines collected in the ZooB small-primate unit were identified after Lent & Wygodzinsky [24]. *Trypanosoma cruzi* infection was first investigated by optical microscopy (OM) in the bugs that arrived alive to the laboratory (see Table 1); fresh (400x) and Giemsa-stained (1000x) hindgut contents were examined. Next, we used a nested PCR (nPCR) to test for *T. cruzi* DNA in triatomine intestinal tissue. DNA was extracted using Illustra tissue and cells genomic Prep Mini Spin Kit (GE Healthcare). We first amplified 188 bp from the *T. cruzi* nuclear repetitive satellite region with primers TCZ1 and TCZ2 [25]; amplicons produced in this PCR were used in a second PCR with primers TCZ3 and TCZ4 [26]; see Additional file 1: Table S1). DNA extracted from a *T. cruzi* culture (Berenice strain, TcII) was used as a positive control, and MilliQ water and DNA from lab-reared, uninfected triatomines as negative controls. PCR products were resolved in 1.3 % agarose gel, stained with ethidium bromide, and visualised using UV fluorescence.

**Table 1:** *Trypanosoma cruzi* infection among *Panstrongylus megistus* collected in a captive-primate unit at Brasília zoo, Federal District, Brazil: bug characteristics and results of optical microscopy and nested PCR

Sex (adults) and stage (nymphs)	Optical microscopy		Nested PCR	
	Tested^a^	Positive^b^	Tested	Positive
Female	12	2	13	11
Male	7	1	7	7
Nymph II	0	-	4	3
Nymph III	8	1	18	16
Nymph V	7	1	8	7
Total	34	5	50	44

^a^:Bugs that arrived dead and dry to the laboratory could not be tested by optical microscopy

^b^:Bugs with a positive optical microscopy were all also positive by nested PCR

### *Trypanosoma cruzi* in primates

Twenty-six Neotropical primates were investigated, six of which were born at ZooB (see Table 2). Blood samples (1 mL) were drawn once (nine specimens) or on two occasions separated by ~24 months (17 specimens) for PCR-based *T. cruzi* detection and quantification. Necropsy samples (intestine, heart, and spleen) from one *Saguinus niger* and one *Callithrix penicillata* that died during the course of the study were also investigated. DNA was extracted from blood samples using the Wizard^TM^ Genomic DNA Purification Kit (Promega), and from necropsy samples using the Mini Spin Plus Kit (Biopur). PCR reactions were first carried out with primers TCZ1 and TCZ2 as described above; the products of this PCR were diluted 1:60 in MilliQ water and 2 μL were used as template for real-time quantitative PCR (qPCR) with Power SYBR© Green chemistry (Applied Biosystems) and primers TCZ3 and TCZ4 [26]; [see Additional file 1: Table S1]. Reactions were run in an ABI 7500 Real-Time PCR System thermocycler (Applied Biosystems) and the results analysed using StepOne v2.3 software (Applied Biosystems). We used MilliQ water and DNA extracted from uninfected mice blood as negative controls. Absolute quantification of parasite DNA was achieved by developing a standard curve with DNA extracted from a Berenice strain *T. cruzi* culture (10^8^ parasites/mL) and serially diluted ten-fold to between 10^5^ and 10^−2^ parasite. The standard curve relates qPCR threshold cycle values and known log-scale DNA concentrations [27, 28]; in our case, theoretical amplification efficiency was ~91 % (slope = −3.6) and the standard curve coefficient of determination was R^2^ = 0.99.

**Table 2:** *Trypanosoma cruzi* infection among captive primates kept at Brasília zoo, Federal District, Brazil: primate characteristics and quantitative real-time PCR (qPCR) results

Number	Species	Origin	State	Year of birth or arrival at ZooB	qPCR^a^	
					First	Second
1	*Alouatta seniculus*	IBAMA	AC	2010	0.000	ND
2	*Aotus nigriceps* ^b^	Born at ZooB	DF	2010	0.003	0.001
3	*Aotus nigriceps* ^b^	Born at ZooB	DF	2012	1.051	0.002
4	*Aotus nigriceps*	IBAMA	AC	2006	0.000	0.000
5	*Aotus nigriceps* ^b^	Born at ZooB	DF	2009	0.000	0.000
6	*Aotus nigriceps*	ND	ND	2007	0.000	0.000
7	*Callicebus cupreus*	AIPU	MA	2006	0.000	0.000
8	*Callithrix geoffroyi*	Jequitinhonha	MG	2010	0.000	0.012
9	*Callithrix penicillata*	IBAMA	DF	2012	0.039	ND
10	*Callithrix penicillata*	IBAMA	DF	2008	0.025	0.001
11	*Callithrix penicillata*	IBAMA	ND	2008	0.029	Dead
12	*Callithrix penicillata*	IBAMA	DF	2012	ND	0.105
13	*Callithrix penicillata*	IBAMA	DF	2012	0.000	0.000
14	*Leontopithecus chrysomelas*	AIPU	MA	2006	0.016	ND
15	*Leontopithecus chrysomelas*	ND	ND	2007	0.000	0.000
16	*Leontopithecus chrysomelas*	Born at ZooB	DF	1999	0.006	ND
17	*Leontopithecus chrysomelas* ^c^	Born at ZooB	DF	2008	0.214	ND
18	*Leontopithecus chrysomelas* ^d^	Born at ZooB	DF	2013	ND	0.007
19	*Leontopithecus rosalia*	AIPU	MA	2010	0.000	0.000
20	*Mico chrysoleucus*	IBAMA	AM	2008	0.025	17.000
21	*Mico argentatus* ^e^	AIPU	MA	2007	0.010	0.006
22	*Pithecia irrorata*	BH zoo	MG	2012	0.073	0.003
23	*Saguinus imperator*	AIPU	MA	2008	0.000	0.000
24	*Saguinus niger*	AIPU	MA	2007	0.003	0.018
25	*Saguinus niger*	ND	PA	2008	0.042	0.001
26	*Saguinus niger*	AIPU	MA	2006	4.000	Dead

^a^:Parasite equivalents/100 ng DNA; First and Second qPCRs were carried out ~24 months apart. ^b^:Born to qPCR-negative mothers (# 4 and 6). ^c^:Born to qPCR-positive mother (# 16). ^d^:Born to qPCR-negative mother (#15). ^e^:Died in 2015. *IBAMA* Instituto Brasileiro do Meio Ambiente e Recursos Renováveis, *ZooB* Brasília zoo, *AIPU* Ararajuba do Ipê primate unit; BH, Belo Horizonte

Brazilian states: *AC* Acre, *AM* Amazonas, *DF* Distrito Federal, *MA* Maranhão, *MG* Minas Gerais, *PA* Pará. *ND* no data/not done

### Identification of *Trypanosoma cruzi* lineages

A fragment of the single-copy nuclear glucose-6-phosphate isomerase (G6pi) gene was PCR-amplified as described in Brenière et al. [29] (see Additional file 1: Table S1), with positive and negative controls as described above for nPCR. Amplicons were analysed in 1.0 % agarose gel, stained with ethidium bromide, and visualised by UV fluorescence. PCR products were purified with the Illustra GFX PCR DNA & Gel Band Purification Kit (GE Healthcare) and submitted to Sanger sequencing. Sequences were edited using Geneious software (Biomatters) and compared with sequences deposited in GenBank using the BLASTn algorithm (http:blast.ncbi.nlm.nih.gov).

## Results

**Fig. 1:**
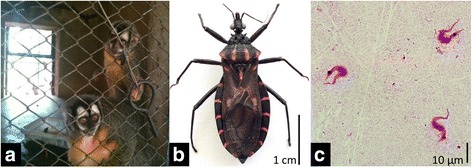
*Trypanosoma cruzi* infection among *Panstrongylus megistus* collected in a captive-primate unit at Brasília zoo, Federal District, Brazil. **a** Captive-primate unit where *P. megistus* specimens were collected. **b** Adult specimen of *P. megistus*. **c** Trypomastigotes detected in *P. megistus* feces after Giemsa staining

We collected 20 adult bugs and 30 nymphs at the ZooB small-primate unit (Table 1, Fig. 1); all were identified as *Panstrongylus megistus*. Thirty-four of those triatomines arrived alive to the laboratory and were examined by OM; five (14.7 %) were found infected with *T. cruzi* (Table 1, Fig. 1). nPCR was positive in the five OM-positive bugs, in 24 OM-negative specimens, and in 15 bugs not examined by OM; thus, overall nPCR positivity was 88 % (Table 1).

*Trypanosoma cruzi* DNA was detected in 17 out of 26 (65.4 %) primates tested by qPCR (Table 2). Qualitative results from blood samples taken ~24 months apart were consistent in 16 out of 17 specimens tested twice; the exception was one *Callithrix geoffroyi* that became qPCR-positive over the course of the study (Table 2). Absolute DNA quantification suggested high parasitaemias in one *Mico chrysoleucus* (17 parasite equivalents/100 ng DNA) and one *Saguinus niger* (4 parasite equivalents/100 ng DNA) (Table 2). Three individuals with *T. cruzi* DNA-positive blood samples died during the study (Table 2); necropsy samples from two of them were submitted to qPCR, which detected small amounts of *T. cruzi* DNA (<1 parasite equivalents/100 ng DNA) in the spleen of one *C. penicillata* and in the heart, spleen, and intestine of the highly parasitaemic *S. niger* mentioned above. Five out of six primates born at ZooB tested positive for *T. cruzi* DNA by qPCR, including three individuals born to qPCR-negative mothers (Table 2).

G6pi sequences from five *P. megistus*, one *S. niger*, and one *M. chrysoleucus* were all 99-100 % identical to that of *T. cruzi* strain OPS21cl11 (TcI lineage, GenBank accession number AY484472.1; see Broutin et al. [30]).

## Discussion

We have presented a detailed description of a *T. cruzi* infection focus in triatomine bugs and captive Neotropical primates at Brasília zoo in central Brazil. Highly-sensitive molecular assays detected *T. cruzi* nuclear DNA in most of the vectors (88 %) and primates (65.4 %) we tested. Infection was identified in primates of six genera and eight species, including the endangered *Leontopithecus chrysomelas*, the vulnerable *Saguinus niger*, and species with unknown preservation status such as *Mico chrysoleucus* and *Pithecia irrorata* (see www.iucnredlist.org). Infection with *T. cruzi* is harmful to the primates and brings about a non-negligible risk of accidental transmission of the parasite to animal keepers, handlers, and veterinarians.

Three primates born to qPCR-negative mothers at ZooB were infected with the same *T. cruzi* strain as *P. megistus* caught in their lodgings. This finding is strongly suggestive of within-cage, *P. megistus*-mediated parasite transmission. Although we did not test for anti-*T. cruzi* antibodies through serology, which might have revealed infection in qPCR-negative individuals [3], the high sensitivity of our qPCR [28] and the rarity of vertical transmission among tamarins [14] make us think that vector-borne transmission was likely the source of most primate infections at ZooB. Primates including humans can acquire *T. cruzi* from triatomines either through direct contact of infected vector faeces with skin or mucosae or by the oral route when bugs carrying the parasite are eaten or contaminate foods or beverages [4, 5, 31]. In addition, that most of the *P. megistus* nymphs we collected inside primate lodgings tested positive for *T. cruzi* (Table 1) clearly implies within-cage transmission of the parasite from infected primates to the vectors – triatomine nymphs lack wings and get *T. cruzi* through infected bloodmeals [24].

Captive-breeding and translocation-reintroduction programs are important for the management (and possibly recovery) of endangered species such as the flagship lion tamarins [32–34]. Preventing or limiting infectious disease spread is one crucial component of such programs [33, 34]. Although *T. cruzi* occurs naturally across the range of all continental American primate species, and although infection with the parasite seems common in many wild populations, the release of infected specimens can be problematic in at least three relevant ways. First, *T. cruzi* is a highly diverse parasite [8, 35], so that foreign strains can be introduced into an area where they do not circulate naturally. Second, *T. cruzi* infection seems to be strongly focal among free-ranging Neotropical primate populations [11, 14]; infected individuals introduced into a low-prevalence site can hence contribute to spreading the parasite. Finally, *T. cruzi*-infected individuals may have relatively low odds of surviving when released into the wild, which may threaten reintroduction success [36]. By showing how high *T. cruzi* infection rates can be among captive Neotropical primates, our results underscore the need to carefully test specimens scheduled for release in the context of endangered-species translocation-reintroduction programs [3, 14].

On a more local scale, we found a thriving *P. megistus* colony within the small-primate unit at ZooB. Infected bugs were found inside primate wooden shelters resembling the hollow-tree vertebrate nests and refuges *P. megistus* occupies in the wild [37]. Although widely distributed, *P. megistus* is primarily associated with the humid Brazilian Atlantic forest [38, 39]; in the Cerrado and other seasonally dry eco-regions, it occurs mainly in moister forest patches. The close proximity of ZooB to a preserved gallery-forest patch where *T. cruzi* (TcI lineage) has been shown to circulate [23] suggests a likely origin for the bugs (and possibly also the parasites) we found. *Panstrongylus megistus*, one of the most important vectors of human Chagas disease, can also colonise in and around human dwellings [9, 24, 37, 38]. Our findings warn about the possibility of domestic or peridomestic, *P. megistus*-borne *T. cruzi* transmission foci in the vicinity of preserved forest patches in Brasília [39] and elsewhere across the Cerrado [37, 38].

Finally, our results highlight the latent risk of accidental *T. cruzi* transmission from infected primates (and probably other mammals) to zoo workers including keepers and veterinarians. If unaware of the infection status of the animals they handle, these workers may be at risk of acquiring the infection while drawing or manipulating biological samples, during surgical or dental procedures, or even when performing necropsies on fresh carcasses. Although needles are involved in most of the accidents reported, *T. cruzi* transmission can also occur through intact mucosae or apparently intact skin, and possibly via droplets or aerosols [40]. Zookeepers may undergo additional risks if they have contact with infected triatomines infesting animal facilities.

## Conclusions

The findings we have presented strongly suggest vector-borne *T. cruzi* transmission within a small-primate unit at Brasília zoo. We suspect that the vectors, and possibly also the parasites, originally came from nearby gallery forest – a hypothesis that can be tested with samples from both habitats and high-resolution molecular markers [35]. In practical terms, we suggest that periodic checks for triatomine infestations and *T. cruzi* infections should become routine practice in captive-animal facilities located near known or suspected vector habitat. Pyrethroid insecticide spraying yields efficient, short-term infestation control; in the long run, the use of bug-refractory animal lodgings – i.e., with fewer potential bug-hiding sites (crevices, cracks…) and easier to inspect and treat – could help prevent or slow re-infestation. Early detection and elimination of *T. cruzi* transmission foci should be of particular interest for captive-breeding and translocation-reintroduction programs involving endangered mammal species. Together with specific training on Chagas disease and *T. cruzi*-related biohazards, this would also help reduce the risk of accidental *T. cruzi* transmission from infected mammals or vectors to captive-animal handlers, keepers, and veterinarians.

## Additional file

Additional file 1: Table S1. PCR conditions and primers used for *Trypanosoma cruzi* detection. (DOC 69 kb)

## Abbreviations

FD: Federal District
G6pi: Single-copy nuclear glucose-6-phosphate isomerase
kDNA: Kinetoplast DNA
PCR: Polymerase Chain Reaction
nPCR: Nested Polymerase Chain Reaction
qPCR: Quantitative real-time PCR
UV: Ultraviolet
ZooB: Brasília zoo.
