# Supplementary material for: Vector-borne transmission of Trypanosoma cruzi among captive Neotropical primates in a Brazilian zoo
Source: Parasit Vectors. 2016 Jan 26;9:39. doi: 10.1186/s13071-016-1334-7 (PMC4727406; doi:10.1186/s13071-016-1334-7)
Supplement: Additional file 1: Table S1. — PCR conditions and primers used for Trypanosoma cruzi detection. (DOC 69 kb) [file 13071_2016_1334_MOESM1_ESM.doc]

**Additional file Table S1 PCR conditions and primers used for *Trypanosoma cruzi*** detection.

| **PCR target** | **PCR code** | **Primer sequence** | **PCR conditions** | **Cycle conditions** | **Fragment length** | | **Reference** | |
| --- | --- | --- | --- | --- | --- | --- | --- | --- |
| Microssatelite repetitive region | TCZ nPCR | TCZ1 (5′- CGA GCT CTT GCC CAC ACG GGT GCT-3′)  TCZ2 (5′-CCT CCA AGC AGC GGA TAG TTC AGG-3′)  TCZ3 (5′- TGC TGC AST CGG CTG ATC GTT TTC GA-3′)  TCZ4 (5´CAR GST TGT TTG GTG TCC AGT GTT GTG A 3´) | 20 ng/μL DNA was used in the first PCR reaction with 2.0 mM MgCl_2_, 0.2μM dNTPs, 0.1μM of TCZ1/2 primers and 1.5U of Platinum Taq DNA polymerase.  2μL of first PCR product was used in nested PCR with 2.0 mM MgCl_2_, 0.2μM dNTPs, 0.2μM of TCZ3/4 primers and 1.5U of Platinum Taq DNA polymerase | First Cycle: 95 ° C /5 min 95 ° C for 30 sec 68 ° C for 1 min 25X 72 ° C for 1 min 72 ° C for 5 min. Second Cycle: 95 ° C /5 min 95 ° C for 40 sec 68 ° C for 40 sec 25X 72 ° C for 30 sec 72 ° C for 7 min. | 188pb  150pb | [25]  [26] | |  |
|  | TCZ qPCR | TCZ1 (see above)  TCZ2 (see above)  TCZ3 (see above)  TCZ4 (see above) | 200 ng/μL DNA was used with 2.5 mM MgCl_2_, 0.2μM dNTPs, 0.2μM of TCZ1/2 primers and 1.5U of Platinum Taq DNA polymerase.  TCZ1/2 products were diluted in 1:60 with MilliQ water and used for real-time PCR with TCZ3/4 primers TCZ3 and TCZ4. Real-time PCR was performed in 20μL reactions, with 10 μL of Power SYBR® Green PCR Master Mix (Applied Biosystems, CA, USA), 0.2 μM of each primer and 2 μL of diluted TCZ1/2 cPCR product. | First Cycle: 95 ° C /5 min 95 ° C for 30 sec 67 ° C for 30 sec 20X 72 ° C for 30 sec 72 ° C for 5 min.  qPCR Cycle: 50 ° C /10 min 95 ° C for 10 min 95 ° C for 15 sec 40X 57 ° C for 1 min 72 ° C for 30 sec | 188pb  150pb | [25]  [26] | |  |
| Single-copy nuclear gene glucose-6-phosphate isomerase | Gpi | GPI-L (5’-CGC CAT GTT GT- GAA TAT TGG-3’)  GPI-R (5’-TTC CAT TGC TTT CCA TGT CA-3’) | Reactions were prepared with 20 ng/μL DNA, 2.0 mM MgCl_2_, 0.2μM dNTPs, 0.4μM of each primer and 1.5U of Platinum Taq DNA polymerase. | Cycle: 94 ° C /3 min 95 ° C for 1 min 58 ° C for 1 min 35 X 72 ° C for 1 min 72 ° C for 5 min. | 652 bp | [29] | |  |
